# Age-associated changes in GSH S-transferase gene/proteins in livers of rats

**DOI:** 10.1080/13510002.2018.1546985

**Published:** 2018-11-16

**Authors:** Shangfu Xu, Dongshun Hou, Jie Liu, Lili Ji

**Affiliations:** a Key Lab for Basic Pharmacology of Ministry of Education and Joint International Research Laboratory of Ethnomedicine, Zunyi Medical University, Zunyi, People’s Republic of China; b Shanghai Key Laboratory of Complex Prescription and MOE Key Laboratory for Standardization of Chinese Medicines, Institute of Chinese Materia Medica, Shanghai University of Traditional Chinese Medicine, Shanghai, People’s Republic of China

**Keywords:** Ontogeny, aging, glutathione S-transferases, rat liver

## Abstract

**Objectives**: Glutathione S-transferases (GSTs) are phase-II metabolic enzymes playing important roles in drug metabolism, anti-oxidative stress and anti-aging. Age is a key factor influencing GSTs expression. Thus, age-related changes of 10 GSTs were examined.

**Methods**: Livers from male Sprague–Dawley rats at fetus (−2 d), neonates (1, 7, 14 and 21 d), puberty (28 and 35 d), adulthood (60 and 180 d), and aging (540 and 800 d), were collected and subjected to qPCR analysis. Liver proteins from 14, 28, 60, 180, 540 and 800 d were also extracted for selected protein analysis by Western-blot.

**Results**: The expression of GSTA1 and GSTP1 increased over the life span and the expression of GSTA4, GSTO1 and GSTZ1 gradually increased until adulthood, and slightly decreased at 800 days. The expression of GSTM1, GSTM3, GSTT1, GSTT2 and GSTK1 gradually increased until adulthood, but significantly decreased during aging of 540 and 800 days. There is a small peak at 7–14 d for GSTA1, GSTP1 and GSTZ1. The protein expression of GSTA1, GSTM1 and GSTP1 followed the trend of mRNA changes.

**Discussion**: This study characterized three expression patterns of 10 GSTs during development and aging in rat liver, adding to our understanding of anti-aging role of GSTs.

## Introduction

Reactive oxygen species (ROS) and electrophiles are highly reactive molecules that are naturally generated in small amounts through metabolism and excessive amounts can damage cellular molecules such as lipids, proteins and DNA [1]. An imbalance in ROS production and adaptive antioxidant capacity results in oxidative stress, leading to cellular dysfunction [2,3]. Organisms have several defense systems to cope with oxygen environment and toxicological stimuli. These defense systems include glutathione systems, antioxidant enzymes and non-enzymatic free radical scavengers. The Nrf2/Keap1 antioxidant pathway constitutes a major detoxification pathway in the body. Activation of Nrf2/Keap1 pathway induces the expression of a panel of genes including the glutathione S-transferases (GSTs) [2].

GSTs are critical phase II enzymes in protecting cellular macromolecules against electrophiles and products of oxidative stress [4]. GSTs are distributed in many tissues, but the liver is the major organ expressing GSTs [5,6]. GSTs can be induced by chemicals and toxicity stimuli, which in turn play an adaptive response to oxidative stress [7].

GSTs are subjected to epigenetic regulation in developing mouse liver [8]. Hepatocyte nuclear factor 4 alpha (HNF4) is proposed to play roles in regulating hepatic mRNA expression of phase II enzymes, including GSTs during development [4]. The glutathione antioxidant system in weaned and young mouse livers is responsible for sensitivity to chemical-induced hepatotoxicity [9]. Indeed, oxidative stress is implicated in many newborn diseases [10], and a better understanding of the antioxidant enzymes during development could inform disease prevention and treatment.

Aging is a physiological process characterized by progressive functional decline in various organs over time. Aging is accompanied by changes in the biotransformation of xenobiotics and impairment of normal cellular functions by free radicals. Aged animals have altered drug-metabolizing and antioxidant enzymes in the livers [11,12]. The higher activity of liver GST in 12-month-old mice might be due to the higher expression of GST mRNA [13] to cope with increased oxidative stress during aging.

We have examined the constitutive expression of age-related drug processing genes changes, from prenatal (−2 d), neonatal (1, 7, 14 and 21 d), at puberty (28 and 35 d), at adulthood (60 and 180 d), and at aging (540 and 800 d), and characterized the age-related expression of kidney transporters [14], hepatic uptake Oatp transporters [15], hepatic efflux transporters [16], and hepatic Nrf2 antioxidant pathways [17]. The goals of this study were to quantify the mRNA abundance of 10 major GST isoforms in rat livers, to detect age-related mRNA and/or protein changes of these isoforms, and to characterize clear age-related patterns from these data.

## Materials and methods

### Animals

Adult male and female SD rats were mated as previously described [14–17]. Positive vaginal plug was considered as Gestation Day 1. Livers of offspring male rats were collected at gestation 19th day (−2 d), at birth (1 d), at the neonatal stage (7, 14, and 21 d), at puberty (28 and 35 d), at the adulthood (60 and 180 d), and at aging (540 and 800 d). All animal experiments were performed in accordance with Chinese Guidelines of Animal Care and Welfare, and approved by the Animal Care and Use Committee of Zunyi Medical University.

### Real-time RT–PCR analysis

Liver tissues were homogenized RNAiso Plus (TaKaRa Biotechnology Co., Ltd., Dalian, China), and real-time RT–PCR was performed as described [17]. Primers were designed by Primer3 and listed in Table 1. Relative expression of genes was calculated by the 2^-ΔΔCt^ method and normalized to the house keeping gene *β*-actin.

**Table 1.** Rat primer sequences used for real-time RT-PCR analysis.

Gene	Accession#	Sequence (5′–3′)		Product length (bp)
		Forward primer	Reverse primer	
*β*-actin	NM_031144.3	ggagattactgccctggctccta	gactcatcgtactcctgcttgctg	150
GST-A1	NM_001024361	aagcaaatatcgccctgatg	tgttcccaacgaggtagtcc	102
GST-A4	NM_001106840	cgggatgctactgacacaga	cggcatacatgtcaatcctg	107
GST-T1	NM_053293	ctgaccatgatccagtgacg	ctagcatgggaaaagggtga	137
GST-T2	NM_012796	gcaatccgtctacaccacct	aaaggggatgccattcttct	123
GST-O1	NM_001007602	tcttatttggccgtggtttc	cggtaggtcttggcatcaat	144
GST-M1	NM_017014	ccacatttttgagcccaagt	tactccattgggccaacttc	149
GST-M3	NM_020540	agaaccaggtcatggacacc	atcttctccgggatggactt	103
GST-P1	NM_012577	tatgccaccgtacaccattg	gagccttgaagccagacatc	129
GST-K1	NM_181371	cacggagtcccagaacattt	tctccctgagcttgctcttc	116
GST-Z1	NM_001109445	cacacatggtgttggagagg	ggaaccggctttaaggtttc	113

### Western-blot analysis

Liver tissues were homogenized in RIPA lysis buffer (Beyotime Institute of Biotechnology, Shanghai, China) containing 1 mM phenylmethanesulfonyl fluoride and freshly prepared proteinase inhibitors. Aliquoted proteins were denatured and separated on NUPAGE 10% BT gels and transferred to PVDF membranes. Membranes were blocked with 5% dry nonfat milk, followed by incubation with primary mouse antibody against *β*-actin, rabbit polyclonal antibodies against GSTA1, GSTM1 and GSTP1 (1:1000) (Biosynthesis Biotechnology Co., LTD. Beijing, China) overnight at 4 °C. After washes with TBST, membranes were incubated with horseradish peroxidase conjugated anti-rabbit, anti-mouse IgG secondary anti-bodies (1:5000) for 1 h at room temperature. Protein antibody complexes were visualized using an Enhanced Chemiluminescent reagent and a ChemiDoc XRS system (Bio Rad Laboratories, Inc., USA). Band intensities were semi-quantified by densitometry using Quantity One® software (version 4.6.2, Bio Rad Laboratories, Inc., USA) [17].

### Statistical analysis

The software SPSS version 16.0 (SPSS, Inc., Chicago, IL, USA) was used for statistical analysis. Data were expressed as the mean ± SEM. Age associated differences were analyzed by one-way analysis of variance, followed by the least significant difference post hoc test. *P *< 0.05 was considered to indicate a statistically significant difference.

## Results

### Age-related mRNA expression of GSTA1 and GSTA4

**Figure 1.**
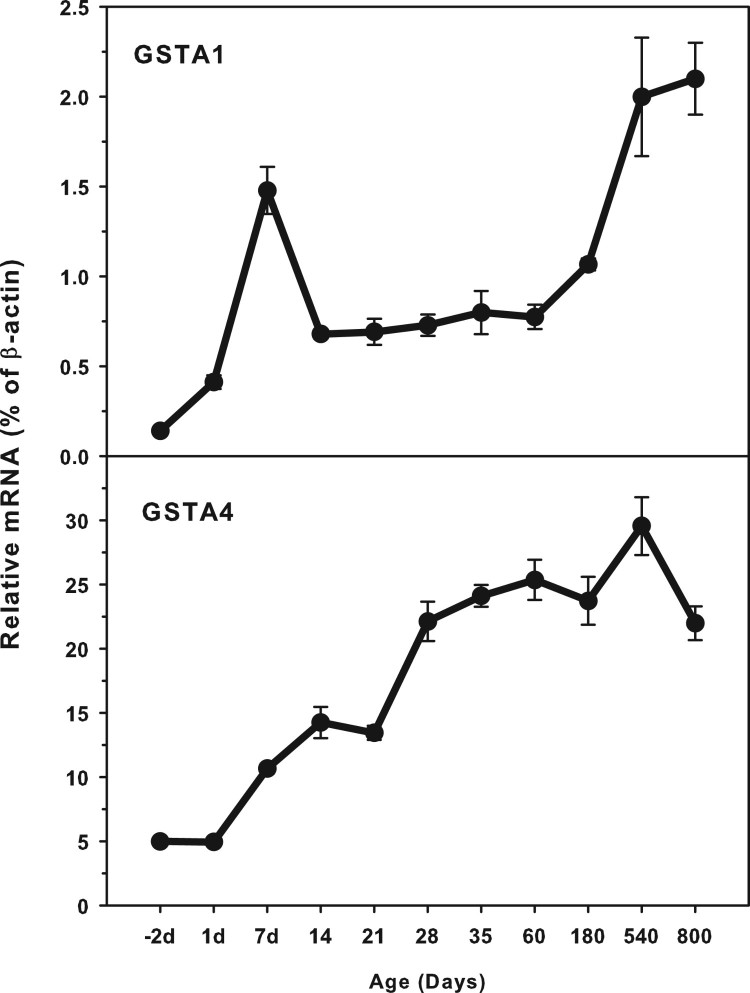
Age-related expression of GSTA1 and GSTA4 in livers of rats. Livers from male SD rats at the fetus (−2 d before birth), the neonatal stage (1, 7, 14 and 21 d after birth), at puberty (28 and 35 d), at adult (60 and 180 d), and at aging (540 and 800 d), were collected to extract RNA. Expression of GST-A1 and GST-A4 was determined by real-time RT-PCR (*n* = 6 for each time point).

The expression of GST A family (GSTA1 and GSTA4) is shown in Figure 1. The highest levels of GSTA4 expression (25.0% *β*-actin) was about 10-fold higher that of GSTA1 (2.5% of *β*-actin). Both GSTA1 and GSTA4 expressed at low levels in the fetus livers, and gradually increased until adulthood, and even higher at aging of 540 and 800 days. There is a peak around 7 days of age for GSTA1. GSTA1 and GSTA4 gradually increased with age.

### Age-related mRNA expression of GSTM1 and GSTM3

**Figure 2.**
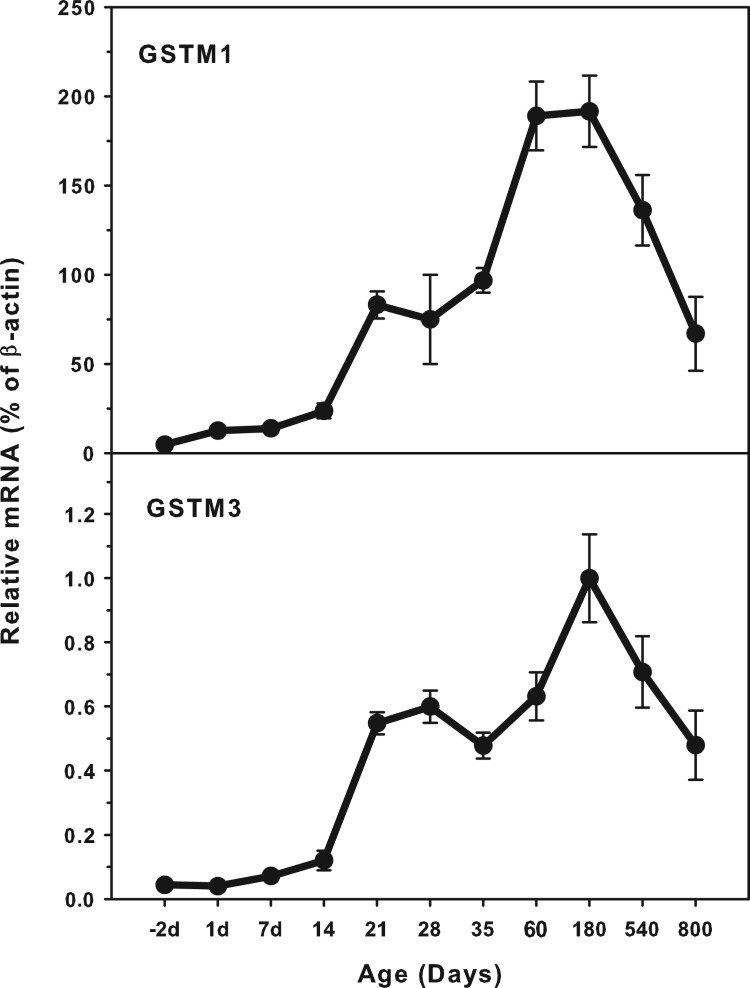
Age-related expression of GSTM1and GSTM3 in livers of rats. Livers from male SD rats at the fetus (−2 d before birth), the neonatal stage (1, 7, 14 and 21 d after birth), at puberty (28 and 35 d), at adult (60 and 180 d), and at aging (540 and 800 d), were collected to extract RNA. Expression of GSTM1 and GSTM3 was determined by real-time RT-PCR (*n* = 6 for each time point).

The expression of GST M family (GSTM1 and GSTM3) is shown in Figure 2. The highest levels of GSTM1 expression (200.0% *β*-actin) was about 20-fold higher that of GSTM3 (1.0% of *β*-actin). Both GSTM1 and GSTM3 expressed at low levels in the fetus livers, and gradually increased till adulthood, but decreased during aging of 540 and 800 days. There is a small peak at weanling of 21 days.

### Age-related mRNA expression of GSTT1 and GSTT2

**Figure 3.**
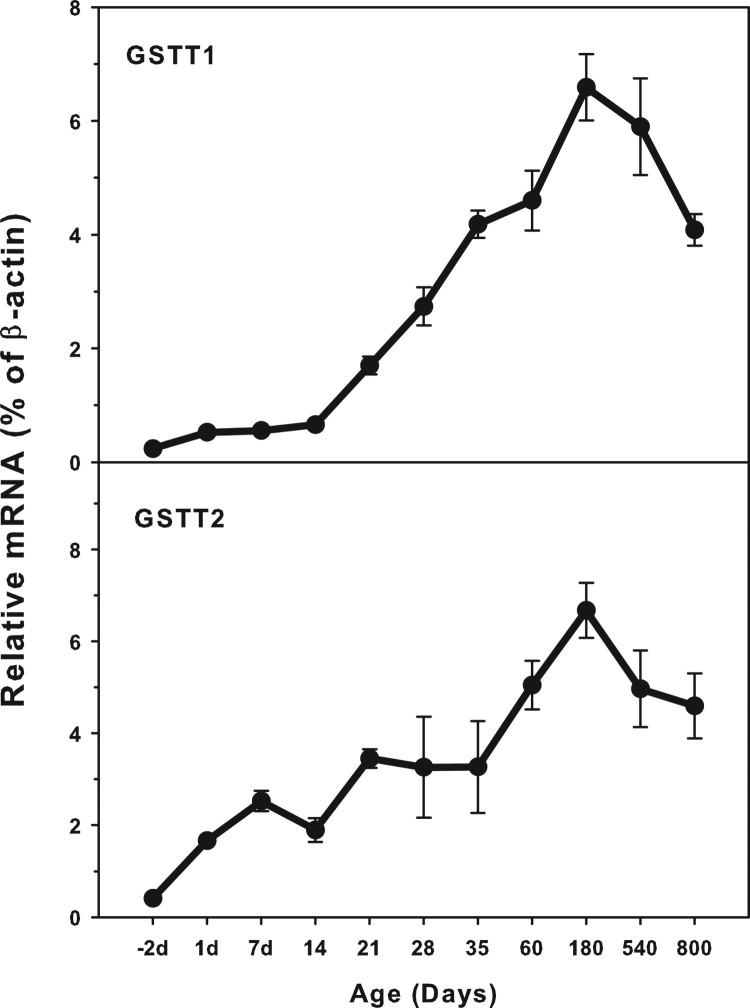
Age-related expression of GSTT1and GSTT2 in livers of rats. Livers from male SD rats at the fetus (−2 d before birth), the neonatal stage (1, 7, 14 and 21 d after birth), at puberty (28 and 35 d), at adult (60 and 180 d), and at aging (540 and 800 d), were collected to extract RNA. Expression of GSTT1 and GSTT3 was determined by real-time RT-PCR (*n* = 6 for each time point).

The expression of GST T family (GSTT1 and GSTT3) is shown in Figure 3. The highest levels of GSTT1 expression (7.0% *β*-actin) was similar to that of GSTT2 (1.0% of *β*-actin). Both GSTT1 and GSTT2 were expressed at low levels in the fetal livers, and gradually increased until adulthood, but both decreased during aging of 540 and 800 days. There is a small peak of GSTT2 at 7 days of age. Both GSTT1 and GSTT2 gradually increased after weaning at 21 days of age.

### Age-related mRNA expression of GSTK1 and GSTO1

**Figure 4.**
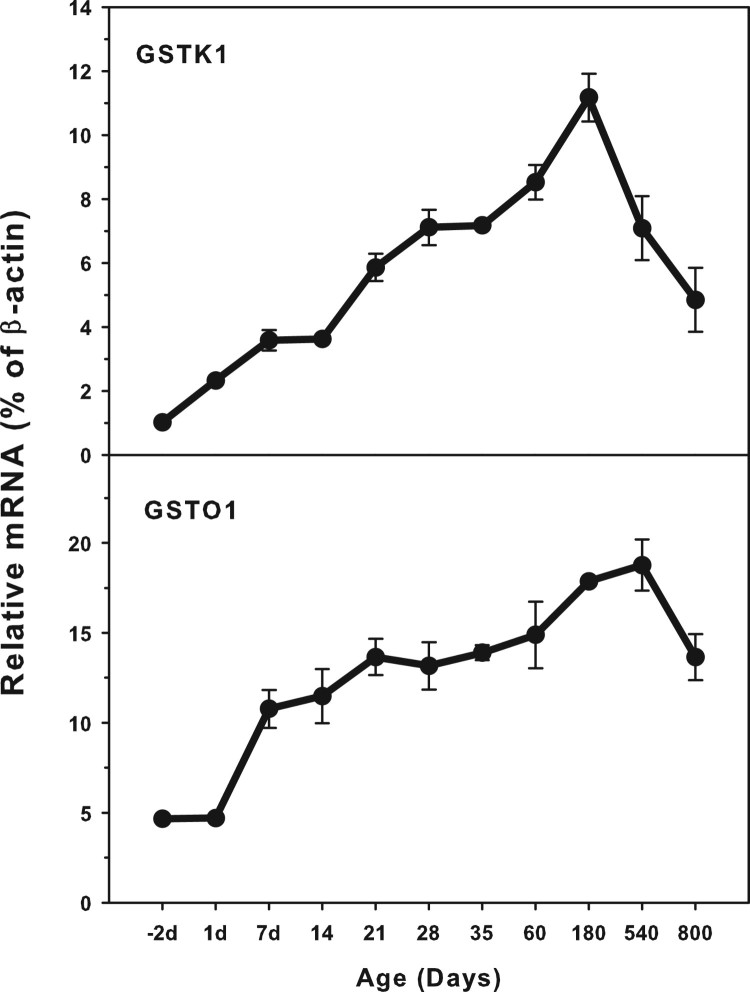
Age-related expression of GSTK1and GSTO1 in livers of rats. Livers from male SD rats at the fetus (−2 d before birth), the neonatal stage (1, 7, 14 and 21 d after birth), at puberty (28 and 35 d), at adult (60 and 180 d), and at aging (540 and 800 d), were collected to extract RNA. Expression of GSTK1 and GSTO1 was determined by real-time RT-PCR (*n* = 6 for each time point).

The expression of GSTK1 and GSTO1 is shown in Figure 4. The highest level of GSTK1 (12.0% *β*-actin) and the highest levels of GSTO1 (15.0% of *β*-actin) were reached. Both GSTK1 and GSTO1 were expressed at low levels in the fetal livers, and gradually increased until adulthood, but both decreased during aging of 540 and 800 days (GSTK1) and 800 days (GSTO1). The expression of both GSTK1 and O1 steadily increased after weaning at 21 days of age until adulthood.

### Age-related mRNA expression of GSTP1 and GSTZ1

**Figure 5.**
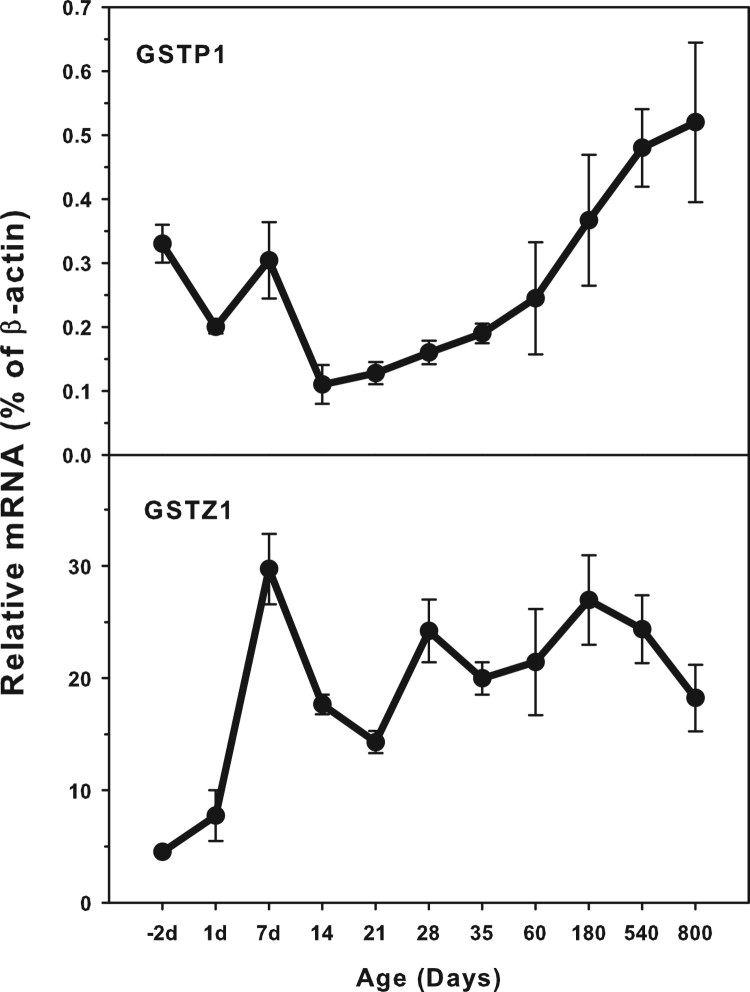
Age-related expression of GSTP1 and GSTZ1 in livers of rats. Livers from male SD rats at the fetus (−2 d before birth), the neonatal stage (1, 7, 14 and 21 d after birth), at puberty (28 and 35 d), at adult (60 and 180 d), and at aging (540 and 800 d), were collected to extract RNA. Expression of GSTP1 and GSTZ1 was determined by real-time RT-PCR (*n* = 6 for each time point).

The expression of GSTP1 and GSTZ1 is shown in Figure 5. The highest levels of GSTP1 expression was 0.6% of *β*-actin at the 800 days of age and the highest levels of GSTZ1 was 30.0% of *β*-actin at 7days of age. GSTP1 expressed at higher levels (0.42% of *β*-actin) in the fetal livers, and gradually decreased until weaning. After weaning it gradually increased until 800 days of age. GSTZ1 expressed low at the fetal livers (5.0% of *β*-actin) and increased rapidly after birth, reaching peak at 7 days of age, and then decreased.

### Age-related protein expression of GSTA1, GSTP1 and GSTM1

**Figure 6.**
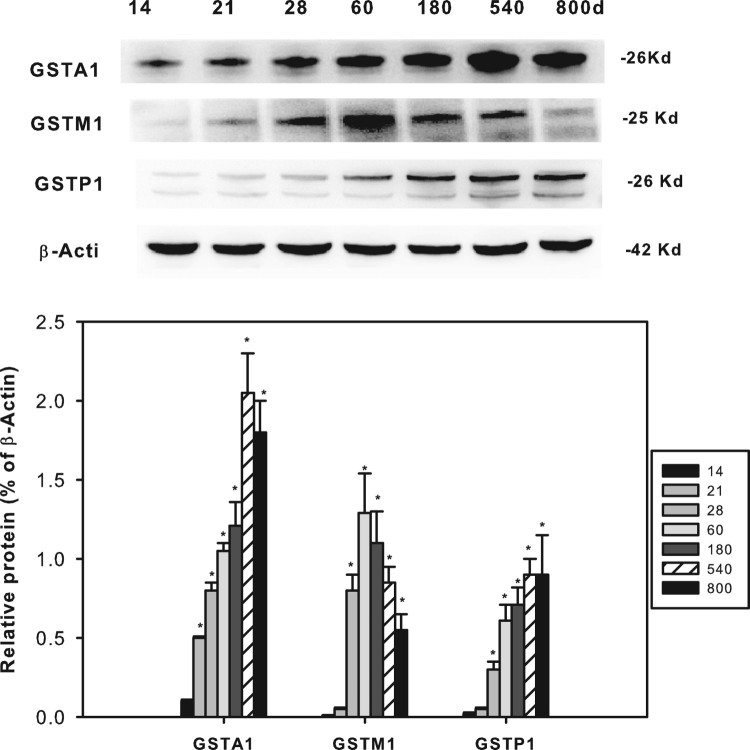
Age-related expression of GSTA1, GSTM1 and GSTP1 in livers of rats. Livers from male SD rats at the neonatal age (14 days of age), at weanling (21 days of age), at adult (60 and 180 d), and at aging (540 and 800 d), were collected to extract protein. Expression of GSTA1, GSTM1 and GSTP1 was determined by western-blots from different blot analysis. Photos shows the representative blots from 3 separate analysis, and the statistical analysis (*n* = 3) was shown in the bottom. *Significant from 14 d, *P *< 0.05.

The protein expression of GSTA1, GSTM1 and GSTP1 is shown in Figure 6. The representative Western blots are shown on the top, and the statistical analyses of 3 separate experiments are shown in the bottom. GSTA1 expression increased after weaning until adulthood (180 d) and aging (540 and 800 d), in agreement with the trend of mRNA analysis; GSTM1 expression also increased after weaning until adulthood (60 and 180 d) but decreased during (540 and 800 d), a trend similar to mRNA analysis; GSTP1 expression increased from 14 days of age and until 800 days of age, also in agreement with the trends of mRNA analysis.

## Discussion

The present study characterized age-related expression of 10 GSTs in livers of rats. There are three patterns of age-related GST expression: (1) the expression of GSTA1 and GSTP1 increased over the life span; (2) the expression of GSTA4, GSTO1 and GSTZ1 gradually increased until adulthood, and slightly decreased at 800 days, and (3) the expression of GSTM1, GSTM3, GSTT1, GSTT2 and GSTK1 gradually increased until adulthood but significantly decreased at 540 and 800 days of age. The mRNA expression of GSTM1, GSTA4, GSTZ1 and GSTO1 in livers was higher, and GSTA1, GSTM1 and GSTP1 proteins followed the mRNA changes. Characterization of GSTs during development and aging in livers of rats could inform studies on drug metabolism and toxicology in children and elderly.

### The alpha class of GSTs

The alpha class of GST is mainly distributed in the stomach, and expression is low in the liver [5]. GSTA1 and GSTA4 can be induced by Nrf2 inducers such as oltipraz, ethoxyquin, and butylated hydroxyanisole, as well as by constitutive androstane receptor (CAR) inducer diallyl sulfide, and pregnane X receptor (PXR) inducer pregnenolone-16-α-carbonitrile (PCN) [7,18]. Using Nrf2-/-, CAR-/-, and PXR-/- mouse models, induction of GST1A1, 1A4 by oltipraz, TCPOBOP, and PCN is largely Nrf2-, CAR- and PXR-dependent [18]. Human GSTA4 shows 53% identity with human GSTA1, and detoxifies the lipid peroxidation product 4-hydroxynonenal (4HNE) [19]. Both GSTA1 and GSTA4 are implicated in liver diseases. For example, mRNA and/or protein levels of GSTA1 and GSTA4 were increased in liver in response to iron overload [20], and in non-alcoholic fatty liver disease (NAFLD) progression [21]. GSTA1 is an earlier and more sensitive indicator of hepatotoxicity of carbon tetrachloride, acetaminophen and ethanol [22]. The importance of GSTA1 and GSTA4 in pharmacology and toxicology promoted researches to characterize their ontogeny and induction [5–7] and the higher activity of liver GST in 12-month-old mice could be an adaptive response to increased oxidative stress during aging [13]. Elevated activities of GST in 21-month-old rats may contribute to protect against xenobiotics as well as electrophiles in aged animals [12]. The present data add to our knowledge of age-dependent changes in alpha class of GST in rats.

### The mu class of GSTs

The mu class of GST consists of at least 6 isoforms. GSTM1 is highest in the liver and also high in the kidneys. GSTM3 is high in the intestine but low in the liver [5]. Using Nrf2-/-, CAR-/-, and PXR-/- mouse models, induction of GSTM1 and GSTM3 by oltipraz, TCPOBOP, and PCN is largely Nrf2-, CAR- and PXR-dependent [18]. Long-term exposure to ethanol induces hepatic GSTM1 and GSTM2, rather than GSTA1 [23]. During the mouse development, GSTM1 and GSTM3 increased with age until puberty [6] and continued to increase during aging before decreasing in old age [11]. The present studies in rats largely agree with these findings in mice, suggesting that the mu class of GSTs presents a pattern distinct from the alpha class.

### The pi class of GSTs

The Pi class of GST consists of GSTP1, GSTP2 and GSTP3. GSTP1 is high in the liver. It is resistant to the induction of microsomal enzyme inducers, and it can be suppressed by PPARα inducers such as clofibrate [7]. Hypermethylation of GSTP1 may play roles in the development of anti-tuberculosis drug-induced liver injury [24]. Epigenetically mediated GSTP1 silencing is associated with enhanced cancer susceptibility and promotes neoplastic transformation allowing cells to acquire additional alterations [25]. During the mouse liver development, GSTP1 increased with age until puberty [6] and continued to increase during aging before decreasing in old age [11]. The present data in rats largely agree with these patterns in mice.

### The theta class of GSTs

The theta class of GST consists of 3 isoforms. GSTT1 is high in the liver; GSTT2 is high in the kidney; and GSTT3 is high in gonads and lungs, and low in the liver [9]. GSTT1 is mainly distributed in the cytosol [26]. Both hepatic GSTT1 and GSTT3 can be induced by PPARα inducers such as clofibrate [7], An association between GSTM1/GSTT1 null mutations and increased risk of anti-tuberculosis drugs has been demonstrated in adults, but not in children [27]. During mouse liver development, GSTT1 and GSTT2 increased with age till puberty [6], and the present study in rats largely agrees with these patterns in mice.

### The kappa, omega, and zeta class of GSTs

GSTK1 is high in the liver, and a mitochondria protein [26]. GSTK1 can be induced by PPARα inducers [7]. Mitochondrial protein levels of GST-K1 were significantly higher in dwarf mice and growth hormone administration downregulated the expression of GSTK1 proteins in dwarf mice [28]. During the development, GSTK1 increased with age till puberty [6], similar to the present observations in rats. GSTK1 continued to increase during adulthood until 180 days of age but decreased markedly at 540 and 800 days of age.

GSTO1 is high in the stomach but is low in the liver [5]. GSTO1 is an important enzyme for hepatic metabolism of arsenic as it catalyzes the cytosolic reduction of DMA(V) in rat liver cytosol [29].

GSTZ1 is the enzyme responsible for conversion of dichloroacetate (DCA) to its inactive metabolites glyoxylate in the liver, and is downregulated in liver cancers [30]. DCA is a mechanism-based inactivator for GSTZ1. The rate of GSTZ1 inactivation by DCA is influenced by age, GSTZ1 haplotype and cellular concentrations of chloride [31]. GSTZ1 has also been implicated in metabolic disorders [32]. The present study shows that GSTZ1 increased during rat liver development, as seen in mice [6], but also demonstrated that GSTZ1 continued to increase until adulthood, and then decrease with.

GSTs are crucial enzymes in anti-aging processes. For example, mitochondrial protein levels of the GSTK1 and GSTM4 are significantly higher in dwarf mice and are regulated by growth hormones [28]; GSTK1 is implicated in the cellular response to internal and external environmental changes, playing a crucial role in the inflammation processes that accompany aging [33]. Aging affects the metabolic capacity of GSTZ1 to detoxify Dichloroacetate (DCA) [34], and GSTs are crucial enzymes in the cell detoxification process catalyzing the nucleophilic attack of GSH on toxic electrophilic substrates and to produce a less dangerous compound, especially by alpha and mu classes of GST [32], and the zeta class of GST received more attention from a therapeutic perspective [31]. Thus, an understanding of age-associated changes of GSTs is of significance in anti-aging research.

## Conclusions

Overall, the present study characterized age-related expression of 10 glutathione S-transferase isoforms, particularly the alpha, mu, and pi classes of GSTs in rat livers during development and aging. GSTs are low in neonatal stages, increase with age, but decreased in aged animals. These data could help our understanding of the effects of GSTs on drug metabolism, pharmacology, and toxicology in the context of aging.
